# A protective role of ciglitazone in ox-LDL-induced rat microvascular endothelial cells *via* modulating PPARγ-dependent AMPK/eNOS pathway

**DOI:** 10.1111/jcmm.12463

**Published:** 2014-11-11

**Authors:** Lei Xu, Shijun Wang, Bingyu Li, Aijun Sun, Yunzeng Zou, Junbo Ge

**Affiliations:** a Shanghai Institute of Cardiovascular Diseases, Zhongshan Hospital, Fudan UniversityShanghai, China; b Institutes of Biomedical Science, Fudan UniversityShanghai, China

**Keywords:** AMPK, eNOS, PPARγ, endothelial cells, LOX-1, angiogenesis

## Abstract

Thiazolidinediones, the antidiabetic agents such as ciglitazone, has been proved to be effective in limiting atherosclerotic events. However, the underlying mechanism remains elucidative. Ox-LDL receptor-1 (LOX-1) plays a central role in ox-LDL-mediated atherosclerosis *via* endothelial nitric oxide synthase (eNOS) uncoupling and nitric oxide reduction. Therefore, we tested the hypothesis that ciglitazone, the PPARγ agonist, protected endothelial cells against ox-LDL through regulating eNOS activity and LOX-1 signalling. In the present study, rat microvascular endothelial cells (RMVECs) were stimulated by ox-LDL. The impact of ciglitazone on cell apoptosis and angiogenesis, eNOS expression and phosphorylation, nitric oxide synthesis and related AMPK, Akt and VEGF signalling pathway were observed. Our data showed that both eNOS and Akt phosphorylation, VEGF expression and nitric oxide production were significantly decreased, RMVECs ageing and apoptosis increased after ox-LDL induction for 24 hrs, all of which were effectively reversed by ciglitazone pre-treatment. Meanwhile, phosphorylation of AMP-activated protein kinase (AMPK) was suppressed by ox-LDL, which was also prevented by ciglitazone. Of interest, AMPK inhibition abolished ciglitazone-mediated eNOS function, nitric oxide synthesis and angiogenesis, and increased RMVECs ageing and apoptosis. Further experiments showed that inhibition of PPARγ significantly suppressed AMPK phosphorylation, eNOS expression and nitric oxide production. Ciglitazone-mediated angiogenesis and reduced cell ageing and apoptosis were reversed. Furthermore, LOX-1 protein expression in RMVECs was suppressed by ciglitazone, but re-enhanced by blocking PPARγ or AMPK. Ox-LDL-induced suppression of eNOS and nitric oxide synthesis were largely prevented by silencing LOX-1. Collectively, these data demonstrate that ciglitazone-mediated PPARγ activation suppresses LOX-1 and moderates AMPK/eNOS pathway, which contributes to endothelial cell survival and function preservation.

## Introduction

Endothelial injury is one of the most common causes of vascular diseases, and is highly associated with the risk factors of atherosclerosis [1–3]. Endothelium-derived nitric oxide is a key molecule in vascular biology, promoting the proliferation of endothelial cells and reducing vascular tone and leukocyte adhesion [4–7]. Nitric oxide bioavailability is mainly regulated by endothelial nitric oxide synthase (eNOS), an enzyme essential to the maintenance of vascular integrity and homeostasis. Oxidative stress, inflammation, smoking, shear stress and diabetes depress the endothelium-dependent vasodilation and cause endothelial cell apoptosis through impairing the synthesis of eNOS [8,9]. The characteristics of elevated blood pressure and reduced vascular repair were observed in eNOS knockout mice [10,11].

In our previous work, we had done a lot to elucidate the role of dendritic cells (DCs) in endothelial injury and atherosclerosis [12–16]. Both oxidized-low density lipoprotein (ox-LDL) and advanced glycosylation end products (AGEs) upregulated the expression of ox-LDL receptor-1 (LOX-1) in atherosclerotic lesions, which led to enhanced accumulation and adhesion of inflammatory cells, including DCs, macrophages and leukocytes in vascular endothelium [14,17]. Ciglitazone and rosiglitazone effectively suppressed DCs maturation, retarded vascular injury, attenuated cell apoptosis and atherosclerosis formation through activating peroxisome proliferator-activated receptor γ (PPARγ) [13,16,18]. However, plenty of studies revealed that PPARγ agonist also improved endotheial function through directly upregulating eNOS activity and nitric oxide synthesis [19–22], but the underlying mechanism was unclear.

Ox-LDL plays a critical role in the atherosclerotic events, not only stimulating maturation and migration of DCs, but also resulting in eNOS uncoupling and decreasing nitric oxide production [23,24]. We previously proved that PPARγ activation could reduce vascular injury through regulating ox-LDL/LOX-1 signalling [13,15,25]. Of interest, ox-LDL mediated atherosclerotic events could also be suppressed by activating AMP-activated protein kinase (AMPK) [26,27], which was a serine/threonine protein kinase, consisting of α, β, γ subunits and highly conserved in animals throughout evolution. AMPK deletion enhanced oxidative injury, reduced eNOS phosphorylation and nitric oxide synthesis [28,29]. However, there is no further evidence regarding to the role of PPARγ agonist in regulating AMPK activity and endothelial function. Therefore, we hypothesized that PPARγ might ameliorate ox-LDL-induced endothelial injury through activating AMPK. In the present study, we investigated the effect of ciglitazone, a common PPARγ agonist, on AMPK/eNOS/nitric oxide pathway and endothelial function.

## Materials and methods

### Reagents

Anti-eNOS (#9572), anti-p-eNOS (#1133), anti-iNOS (#2982), anti-Akt (#4691), anti-p-Akt (#4060), anti-AMPK (#2532) and anti-p-AMPK (#2531) were purchased from Cell Signalling Technology (Beverly, MA, USA); anti-PPARγ (sc-7273) were purchased from Santa Cruz Biotechnology (Santa Cruz, CA, US); anti-LOX-1 (ab60178) were purchased from Abcam, plc (Cambridge, UK); Ciglitazone and GW9662 were purchased from Sigma-Aldrich Inc., St. Louis, MO, USA; and ox-LDL were purchased from Beijing XieSheng Biotechnology Limited-liability Company (Beijing, China).

### Cell culture

Eight weeks old male Wistar rats were chosen for the present study. After anaesthetized by intraperitoneal injection of 1% pentobarbital sodium (1 ml/100 g), rats were killed and the chest was opened to expose the heart, the heart was isolated from the ascending aorta, and quickly moved to the D-Hanks equilibrium salt buffer (0.01 M, pH 7.3). After washing the chambers of heart for three times, the LV tissue was cut and minced to small pieces (∼2 mm^3^). These small tissues were uniformly placed onto the Petri dishes with 10 cm diameter, which containing the foetal bovine serum (FBS), and cultured with a condition of 37°C, 5% CO_2_ for 2 hrs, then changed with high glucose DMEM containing 20% FBS. From 48 to 72 hrs after culturing, the cells were proliferated and separated from the stick tissues, and then the tissues were carefully removed. The cells were digested by 0.125% pancreatin trypsin with 0.02% EDTA, and the 2–3 generation of cells were used in the present study. Cells were induced by for 24 to 72 hrs.

### Small interfering RNA transfection

siRNA targeting AMPK (accession number NM_019142), PPARγ (accession number NM_001145367), LOX-1 (accession number NM_133306) and non-specific scramble RNA were synthesized by Genepharma (Shanghai, China). The siRNA sequences used were listed in Table 1. Transfection was done by Lipofectamine2000 reagent (Invitrogen, Carlsbad, CA, US), following the manufacturer's instructions. Transient transfection was performed on RMVECs for 24 hrs, and then the medium was switched to DMEM containing 1% FBS for another 6 hrs. Cells were then stimulated by ox-LDL (50 μg/ml). At the end of each experiment, Loss-of-Function of respective proteins was assessed by Western blotting.

**Table 1:** The sequences of siRNA designed for genes AMPKα1, PPARγ and LOX-1 are listed

Gene	siRNA sequence
AMPKα1 NM_019142	S 5′-GGGUAACAUUAUGAACUAUtt-3′
	AS 3′-ttCCCAUUGUAAUACUUGAUA-5′
PPARγ NM_001145367	S 5′-GUUCCUUCUAUCGAUUGCAtt-3′
	AS 3′-ttCAAGGAAGAUAGCUAACGU-5′
LOX-1 NM_133306	S 5′-CCAUUAUGCUAGAGGUAAUtt-3′
	AS 3′-ttGGUAAUACGAUCUCCAUUA-5′

### Real-time PCR

Total RNA was extracted from RMVECs using One Step PrimeScript® RT-PCR Kit II (TaKaRa Biotechnology, Dalian, China) with provided methods by the manufacturer. Quantitative real-time PCR was performed on Rotor-Gene 3000 (Corbett Research, New South Wales, Australia) according to the condition of 95°C for 10 sec., 55°C for 15 sec. and 72°C for 15 sec. for 30 repeated cycles. The primers used as listed in Table 2. All the gene expressions were detected and normalized to GAPDH, the levels of control or so-called ‘housekeeping’ gene. The relative changes in gene expression were analyzed by the 2^−ΔΔCt^ method.

**Table 2:** The sequences of mRNA primers designed for genes eNOS and LOX-1 are listed

Gene	Primer sequence
eNOS NM_021838	S 5′-CTGTATGGCTCTGAGACTGGC-3′
	AS 3′-CGTGTTCTCAATGTTTTAGGCTA-5′
LOX-1 NM_133306	S 5′-TATCAGTGACCCTTATTGTAC-3′
	AS 3′-TTCTTAGTTTCTCCCTTGA-5′

### Western blotting

RMVECs were extracted in lysis buffer (10 mM Tris-HCl, pH 8, 140 mM NaCl, 5 mM EDTA, 0.025% NaN_3_, 1% Triton X-100, 1% deoxycholate, 0.1% SDS, 1 mM PMSF, 5 μg/ml leupeptin, 2 μg/ml aprotinin, 50 mM NaF and 1 mM Na_2_VO_3_). Equal amount of total proteins (30 μg) were separated on 12% SDS-PAGE and transferred to a PVDF membrane (Millipore Corp., Bedford, MA, US). The membrane blots were first blocked by 5% bovine serum albumin for 1 hr, and then incubated with the primary antibody at 4°C overnight. After washing with PBS for 3 times, the blots were incubated by anti-rabbit or anti-mouse immunoglobulin-G conjugated with horseradish peroxidase at room temperature for another 2 hrs. The immunoreactive proteins were visualized and quantified by the ChemiDoc™ system (Bio-Rad, Hercules, CA, US).

### Cell apoptosis analysis by TUNEL assays

*In vitro* cultured RMVECs were stimulated by ox-LDL for 24 hrs, cell apoptosis was examined using the terminal deoxynucleotidyltransferase-mediated dUTP nick end labelling (TUNEL) assay (In Situ Cell Death Detection Kit, Roche Applied Science, IN, US). TUNEL labelling was performed according to the manufacturer's protocol. Quantification of Apoptotic Index (AI) was determined by counting TUNEL positive cells from 10 random fields per section and was expressed as a percentage of total cells.

### Endothelial tube formation

*In vitro* cultured RMVECs (0.5 × 10^5^ cells) were detached, resuspended and layered onto 24-well plates containing growth factor-reduced Matrigel matrix (200 μl; BD System, Franklin Lakes, NJ, US) which allowed to solidify before at 37°C. Cells cultivated at 37°C with 5% CO_2_ /95% O_2_ for 24 hrs. Capillary-like tube formation was captured by a digital output camera attached to an inverted phase-contrast microscope and analyzed by software systems (Leica, Wetzlar, Germany).

### Isometric measurements of vascular function

Vascular function was studied by measuring the isometric force of aortic rings in organ bath as previously described [30]. After anesthetized by intraperitoneal injection of 1% pentobarbital sodium (1 ml/100 g), rat hearts were rapidly removed and aortic rings (2 mm in length) was excised and rinsed in cold physiological saline solution, then the aortic rings were mounted in organ chambers for recording changes of isometric tension. Rings were preload to a basel tension of 0.5 g force and allowed to equilibrate in Krebs solution for 45 min. The rings were first exposed to maximally effective concentration with phenylephrine (5 μmol/l), and then acetylcholine (ACh) was added cumulatively to induce endothelium-dependent relaxation. Dose-response curves were recorded by the cumulative addition of ACh (10 nmol/l to 10 μmol/l).

### Assessment of cellular nitric oxide production

RMVECs were incubated with ox-LDL (50 μg/ml) and with different indicative stimulus. At the end the experiment point, cytoplasm was collected and 300 μl for each sample was required for sequence coincubation with 200 μl agent A and 200 μl agent B (nitric oxide assay kit provided, NanJing Jiancheng Corporation) for 10 min., and centrifugation at 4400 g for 15 min. The supernatant was mixed with the chromogenic agent C (80 μl) for 15 min., finally the OD value for each sample was examined by microplate reader at 550 nm with a peak absorption.

### Statistical analysis

Data are presented as mean ± SEM. Statistical analyses were performed by two-tailed indirect Student's *t*-test or one-way anova with a post-hoc least significant difference test using SPSS 15.0 software (http://www-01.ibm.com/software/analytics/spss/). Differences were considered significant at a *P* < 0.05 level.

## Results

### Ciglitazone reversed eNOS expression in RMVECs induced by ox-LDL

**Fig. 1:**
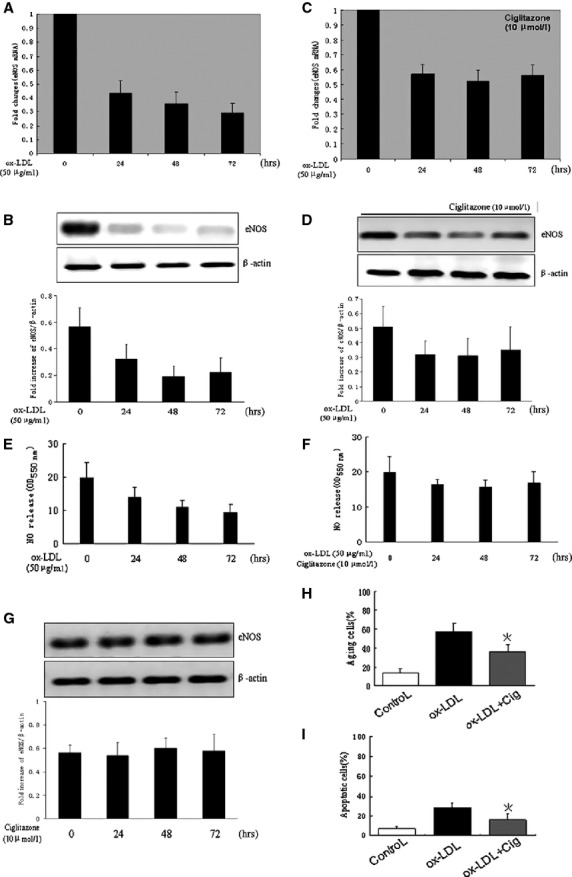
Ciglitazone enhanced eNOS expression in ox-LDL-induced RMVECs. *In vitro* cultured RMVECs were stimulated by ox-LDL (50 μg/ml) for 0, 24, 48 and 72 hrs with or without ciglitazone (10 μmol/l) pre-treatment. (**A** and **B**) The mRNA and protein levels of eNOS were detected by real-time PCR and Western blotting. (**C** and **D**) eNOS expression with pre-treatment of ciglitazone and (**G**) in the absence of ox-LDL. (**E** and **F**) Relative quantification of nitric oxide production using nitrate reductase method in RMVECs pre-treated with or without ciglitazone. (**H** and **I**) The percentage of ageing and apoptotic cells detected by TUNEL staining and cell counting. *P < 0.05 *versus* ox-LDL-induced group (*n* = 5 separated experiments).

We first investigated the role of ciglitazone in ox-LDL-induced endothelial injury. RMVECs were stimulated by ox-LDL (50 μg/ml) for various times, including 24, 48 and 72 hrs, in the presence or absence of pre-treatment with ciglitazone (10 μmol/l). Both mRNA and protein levels of eNOS were examined in RMVECs using real-time PCR and Western blotting. In a time-dependent manner, ox-LDL significantly suppressed eNOS expression by about 60% of mRNA, and more than 50% of protein at 24 hrs after stimulation (Fig. 1A and B). However, downregulation of eNOS, the transcriptional and post-transcriptional levels, was significantly reversed by ciglitazone pre-treatment for 24 hrs (Fig. 1C and D), but ciglitazone had no effect on the basal eNOS production in the absence of ox-LDL (Fig. 1E). Considering that endothelial function was mostly dependent eNOS phosphorylation and nitric oxide synthesis, and Akt/eNOS pathway was critical for endothelial cell-mediated angiogenesis, we further investigated the impact of ciglitazone on regulation of the activities of eNOS and Akt. Phosphorylation of eNOS on Ser^1177^ was reduced by ox-LDL in RMVECs, but significantly enhanced by ciglitazone even in the presence of ox-LDL stimulation (Fig. S1). In addition, our data showed that nitric oxide was time-dependently reduced by ox-LDL induction, which was prevented by ciglitazone pre-treatment (Fig. 1F), suggesting that ciglitazone-mediated production was largely dependent on the phosphorylation of eNOS. Similarly, ciglitazone also increased activity by enhancement of Akt phosphorylation on Ser^473^, but ciglitazone had little effect on baseline Akt activity (Fig. S2). Accordingly, the transcriptional level of VEGF in RMVECs was also strongly increased by ciglitazone in response to ox-LDL, but treatment with ciglitazone alone had little effect on VEGF mRNA expression (Fig. S3). Collectively, these data suggested that Akt/VEGF, the aspect of angiogenic signalling might be indirectly regulated by ciglitazone.

Nitric oxide synthesis not only improved cell survival, but also induced cell apoptosis, ageing and even cell death. Previous study showed that iNOS/nitric oxide pathway played a critical role in cell apoptosis [31], we therefore examined the iNOS expression in RMVECs, however, baseline level of iNOS was about fivefold lower than eNOS (Fig. S4), and induction with ciglitazone failed to upregulate the iNOS expression in RMVECs. Furthermore, apoptosis and ageing were comparably lower in resting RMVECs, but the number of apoptotic and ageing cells were both remarkably increased after induction with ox-LDL for 24 hrs, and this effect could be reversed by ciglitazone pre-treatment (Fig. 1G and H), indicating that ciglitazone-mediated eNOS signalling played a major role in improving RMVECs survival rather than inducing cell apoptosis.

### AMPK activation was essential to ciglitazone-mediated eNOS signalling

**Fig. 2:**
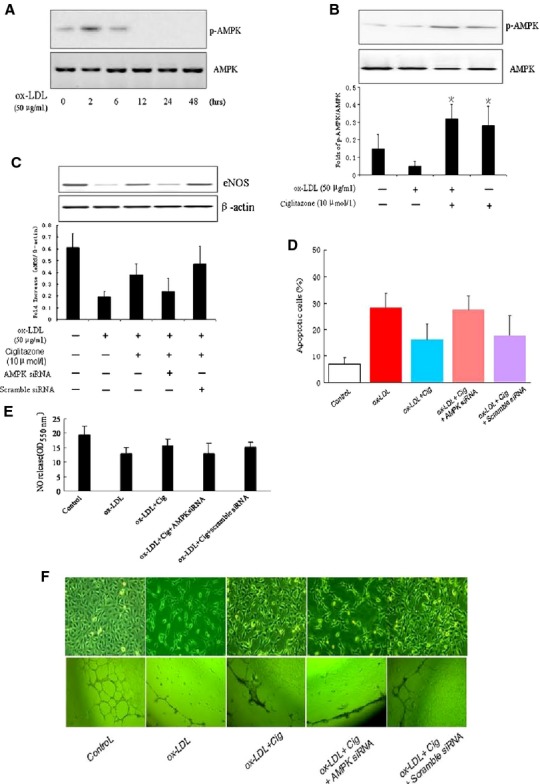
AMPK activation was required for ciglitazone-induced eNOS expression. (**A**) *In vitro* cultured RMVECs were induced by ox-LDL (50 μg/ml) in a time-dependent manner, the phosphorylation level of AMPK was detected by Western blotting. (**B**) AMPK phosphorylation in RMVECs after induction with ox-LDL (50 μg/ml) for 24 hrs with or without ciglitazone (10 μmol/l) pre-treatment. (**C**) The effect of ciglitazone (10 μmol/l) on ox-LDL-induced eNOS expression with or without AMPK siRNA (and scramble siRNA as negative control). (**D**) The effect of ciglitazone (10 μmol/l) on ox-LDL-induced RMVECs apoptosis with or without AMPK siRNA (scramble siRNA as negative control) detected by TUNEL staining. (**E**) Relative quantification of nitric oxide production using nitrate reductase method in RMVECs under different treatments. (**F**) Representative images of cell ageing (up) and endothelial tube formation in Matrigel Matrix from ox-LDL-induced RMVECs with or without pre-treatment of ciglitazone and AMPK siRNA (scramble siRNA as negative control). *P < 0.05 *versus* Control group; ^#^*P* < 0.05 *versus* ox-LDL treated group; ^§^*P* < 0.05 *versus* ox-LDL and ciglitazone treated group (*n* = 5 separated experiments).

Previous studies indicated an important role of AMPK in regulating the expression of eNOS [28,32,33]. Therefore, we investigated whether AMPK was also involved in ox-LDL-mediated suppression of eNOS, and whether AMPK activity was affected by ciglitazone treatment. AMPK phosphorylation was examined in ox-LDL-stimulated RMVECs from 0 to 48 hrs. The results showed that the level of phosphorylated AMPK was greatly attenuated by ox-LDL at 6 hrs after ox-LDL (Fig. 2A). However, the inhibitory effect of ox-LDL on AMPK activity was significantly reversed by pre-treatment cells with ciglitazone, and ciglitazone also induced an increase of AMPK phosphorylation in the absence of ox-LDL (Fig. 2B), suggesting a critical role of ciglitazone in activating the baseline of intercellular AMPK activity. Next, we knocked down AMPK gene in RMVECs by transient transfection of specific siRNA, and we further examined the impact of AMPK silencing on eNOS expression. As expected, the eNOS expression was suppressed by ox-LDL, and re-enhanced by pre-treatment with ciglitazone. Of interest, ciglitazone-induced upregulation of eNOS was abolished by transfecting with AMPK siRNA in RMVECs compared with that transfecting with scramble siRNA (Fig. 2C). Considering that eNOS phosphorylation and nitric oxide synthesis were upregulated by ciglitazone, we tested the role of ciglitazone in endothelium-dependent relaxation (EDR) after ox-LDL stimulation. As shown in supplement Figure 4, although EDR in response to Ach was not significantly reduced in aortic segments incubated with ox-LDL(50 μg/ml) for 1 hr when compared with control, it manifested slight improvement numerically after preatment with ciglitazone for 30 min., and the proportion of vaso-relaxation decreased with AMPK inhibitor compound C (5 μmol/l), indicating an important role of AMPK in endothelial function.

Furthermore, depletion of AMPK failed to prevent increased RMVECs ageing and apoptosis induced by ox-LDL, even in the presence of ciglitazone pre-treatment (Fig. 2D and F up line). Because AMPK inhibition impaired EDR, we further detected this effect on nitric oxide synthesis in RMVECs. As expected, ciglitazone-mediated nitric oxide production in ox-LDL-induced RMVECs was blunted by pre-treating cells with AMPK siRNA (Fig. 2E).

We next examined whether RMVECs-dependent angiogenesis was also affected by knocking down AMPK because Akt/VEGF signalling was regulated by ciglitazone (as shown in Fig. S2 and S3). The results showed that RMVECs-mediated angiogenesis was blunted by ox-LDL (50 μg/ml), but significantly reversed by pre-treating cells with ciglitazone (10 μmol/l). Interestingly, ciglitazone-induced endothelial tube formation in Matrigel Matrix was inhibited by transfecting AMPK siRNA in RMVECs (Fig. 2F). Taken together, these data indicated that AMPK was critically involved in ciglitazone-mediated angiogenesis, EDR and protection of RMVECs against ageing and apoptosis.

### Ciglitazone-mediated AMPK/eNOS pathway was PPARγ dependent

**Fig. 3:**
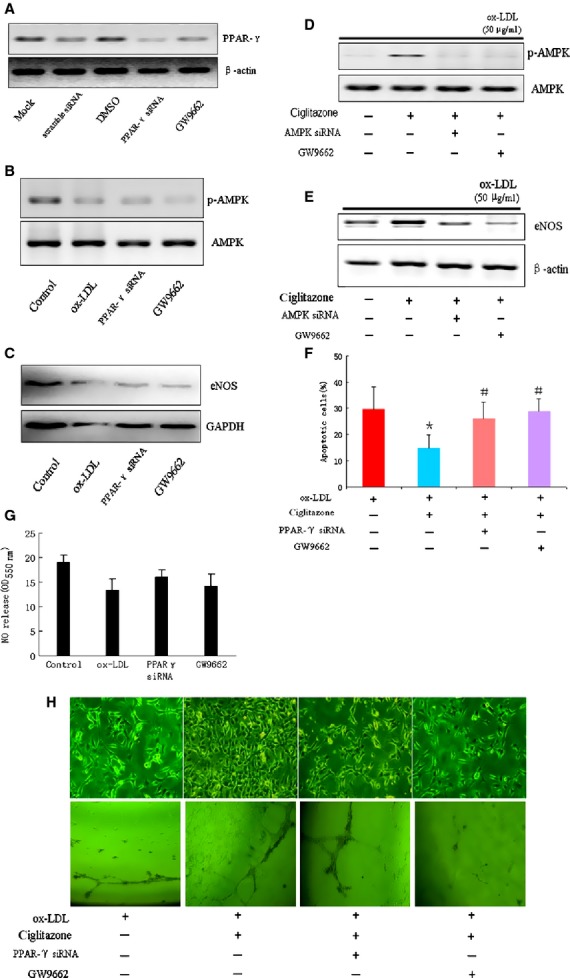
PPARγ activation was required for ciglitazone-induced AMPK/eNOS signalling transduction. (**A**) PPARγ expressions were detected in RMVECs pre-treated with PPARγ siRNA transfection (and scramble siRNA as negative control), or pre-treated with GW9662 (and DMSO as negative control). (**B** and **C**) The expressions of AMPK phosphorylation and eNOS were detected in RMVECs induced by ox-LDL, PPARγ siRNA, or GW9662. (**D** and **E**) The effect of ciglitazone (10 μmol/l) on ox-LDL-induced AMPK phosphorylation and eNOS expression after pre-treated with PPARγ siRNA or GW9662. (**F**) The effect of ciglitazone (10 μmol/l) on ox-LDL-induced RMVECs ageing and apoptosis after pre-treated with PPARγ siRNA or GW9662. (**G**) Relative quantification of nitric oxide production using nitrate reductase method in RMVECs under different treatments. (**H**) Representative images of cell ageing (up) and endothelial tube formation in Matrigel Matrix from ox-LDL-induced RMVECs with or without pre-treatment of ciglitazone, PPARγ siRNA and GW9662. *P < 0.05 *versus* Control group; ^#^*P* < 0.05 *versus* ox-LDL treated group (*n* = 5 separated experiments).

Considering that PPARγ ligands had been previously reported to increase expression of eNOS and nitric oxide synthesis in endothelial checked cells *in vivo* and *in vitro* [18,22,34], we next tested whether ciglitazone-mediated AMPK activation and eNOS expression was PPARγ dependent. RMVECs were pre-treated with PPARγ siRNA and GW9662 (a special PPARγ antagonist), respectively for 24 hrs, the expression of PPARγ was verified by Western blotting. PPARγ was significantly reduced in RMVECs by either siRNA transfection or GW9662 stimulation, when compared with scramble siRNA or DMSO treated RMVECs (Fig. 3A). Importantly, we observed that both PPARγ siRNA and GW9662 pre-treatment could significantly suppress the phosphorylation levels of AMPK, and both also led to a remarkable reduction of eNOS in RMVECs, when compared with the effect of ox-LDL (Fig. 3B and C). Next, we examined the effect of PPARγ inhibition on ciglitazone-induced AMPK activity and eNOS expression. Data showed that the protective effect of ciglitazone on AMPK activation and eNOS expression was depleted by inhibiting PPARγ with PPARγ siRNA or GW9662 (Fig. 3D and E), indicating an essential role of ciglitazone-induced PPARγ in regulating AMPK/eNOS signalling. In addition, PPARγ siRNA or GW9662 treatment also suppressed the nitric oxide production in RMVECs (Fig. 3G). Although ox-LDL-induced cell ageing and apoptosis was decreased by ciglitazone pre-treatment, this protective effect was abolished by suppressing PPARγ with specific siRNA or GW9662 (Fig. 3F and H). Notably, ciglitazone-mediated angiogenesis was also significantly reduced by PPARγ siRNA or GW9662 (Fig. 3H).

### PPARγ dependent AMPK activation suppressed LOX-1 expression in RMVECs

**Fig. 4:**
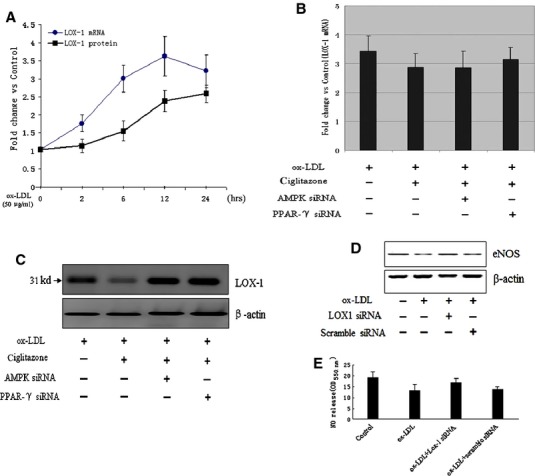
PPARγ-dependent AMPK activity enhanced eNOS expression through regulating LOX-1 receptor. (**A** and **B**) The effect of ciglitazone (10 μmol/l) on ox-LDL-induced LOX-1 expression in RMVECs after pre-treated with PPARγ siRNA or GW9662. (**C**) The effect of ciglitazone (10 μmol/l) on ox-LDL-induced LOX-1 protein levels with or without AMPK siRNA or PPARγ siRNA, respectively. (**D**) The effect of LOX-1 siRNA on ox-LDL-induced eNOS expression in RMVECs. (**E**) Relative quantification of nitric oxide production in ox-LDL-induced RMVECs with or without pre-treatment of LOX-1 siRNA.

LOX-1 was one kind of newly identified ox-LDL receptor in endothelial cell surface, which played a critical role in endothelial dysfunction and development of atherosclerosis. Recent studies suggested that ox-LDL suppressed eNOS expression was mainly *via* LOX-1 dependent oxidative stress [35–37], and LOX-1 inhibition promoted the upregulation of eNOS [38]. To investigate whether ciglitazone protected *in vitro* cultured RMVECs from ox-LDL injury through downregulating LOX-1 expression, we examined the mRNA and protein levels of LOX-1 in ox-LDL-induced RMVECs after pre-treating cells with ciglitazone for 24 hrs. The results showed that both mRNA and protein levels of LOX-1 were significantly enhanced after ox-LDL stimulation, but only the protein of LOX-1 was suppressed by ciglitazone induction (Fig. 4A and B), suggesting LOX-1 expression was regulated by ciglitazone in part through post-transcriptional control. Notably, ciglitazone-mediated reduction of LOX-1 protein in RMVECs was abrogated by AMPK siRNA and PPARγ siRNA, respectively, followed by stimulation with ox-LDL for 24 hrs (Fig. 4C). Importantly, both Akt phosphorylation and eNOS expression were suppressed by ox-LDL-induced RMVECs, which were significantly reversed by inhibition of receptor signalling with special siRNA against LOX-1 (Fig. 4D and Fig. S7). Inhibition of LOX-1 also significantly increased the nitric oxide production in RMVECs which was suppressed by ox-LDL (Fig. 4E), suggesting that LOX-1 was essential for ox-LDL-mediated inhibition of Akt and eNOS activity, and downstream nitric oxide synthesis.

## Discussion

**Fig. 5:**
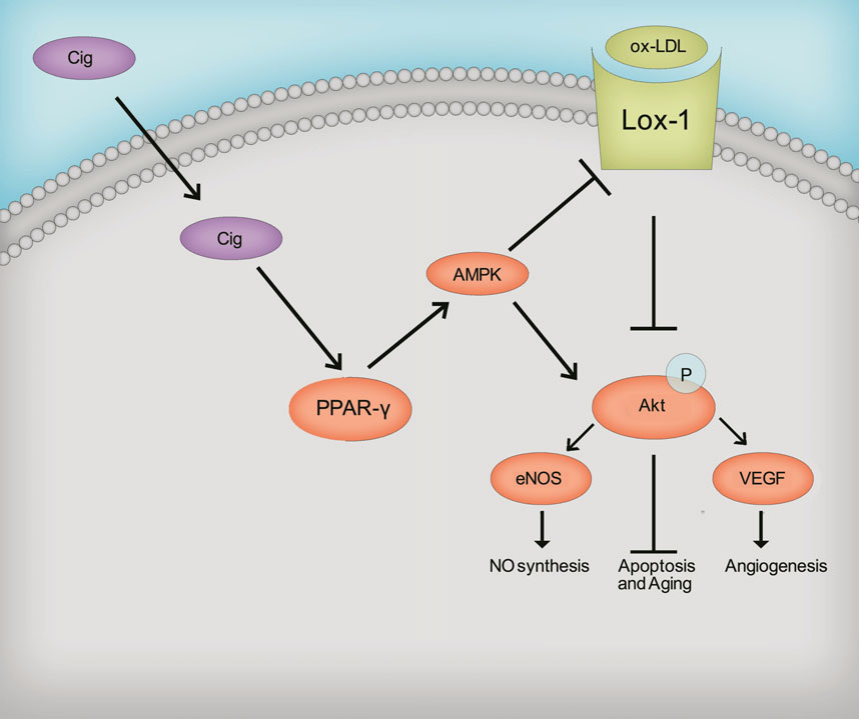
A model of possible mechanism of ciglitazone-mediated protection on endothelial cells against ox-LDL-induced injury through activating PPARγ-dependent AMPK signalling pathway.

ox-LDL played an important role in the progression of atherosclerosis through receptor endocytosis-mediated foam cell formation and inflammatory and oxidative responses. Accumulated endocytosis of ox-LDL led to the injury of endothelium and resulted in atherogenesis. Recent studies revealed a central role of eNOS in the maintenance of endothelial function and integrity, and PPARγ ligands such as 15d-PGJ_2_ and ciglitazone could increase nitric oxide release in endothelial cells [39,40]. However, the underlying mechanism for the protective role of ciglitazone in endothelium and vascular homeostasis remained not well defined. In this study, we provided the evidence that (*i*) ciglitazone protected *in vitro* cultured RMVECs by reducing cell apoptosis and ageing; (*ii*) ciglitazone promotes nitric oxide synthesis through activating Akt and upregulating eNOS expression; (*iii*) ciglitazone improves angiogenesis through Akt-dependent VEGF signalling and (*iv*) PPARγ-dependent AMPK phosphorylation induced by ciglitazone downregulates the ox-LDL receptor, LOX-1, and enhances Akt and eNOS activities, thereby further enhanced nitric oxide synthesis (Fig. 5).

AMPK, a serine/threonine protein kinase, was critically involved in cellular energy metabolism. AMPK activation could suppress the oxidative stress *via* reducing mitochondrial ROS production [28], and therefore contributed to mitochondrial protection homeostasis of endothelial function [26,41]. Recent studies further suggested a critical role of AMPK in stabilizing the endothelial function through regulating eNOS signalling [28,29,32,33]. Metformin, the AMPK activator, proved to be effective in meditating vascular protection against calcification, improving myocardial infarction and attenuating ventricular hypertrophy, all of which required the mechanism of AMPK-dependent eNOS activation [26,29,42,43]. Recent studies revealed that Metformin can reduce LOX-1 expression in endothelial cells, which might contribute to attenuation of endothelial injury, vascular calcification and improvement of angiogenesis [17,44,45]. Our data further confirmed the findings of previous studies. In addition, we found that AMPK activity could be enhanced by ciglitazone, and both eNOS expression and activity suppressed by time-dependent ox-LDL stimulation was re-enhanced after ciglitazone treatment, but such effects were abolished by AMPK inhibition. Notably, inhibition of eNOS activity by its associated protein, such as HSP90, would enhance the proteolytic degradation of the enzyme [46–48]. Thus, ciglitazone might stabilize eNOS expression *via* promoting eNOS phosphorylation and counteract ox-LDL-induced eNOS uncoupling. We confirmed that eNOS phosphorylation on Ser^1177^ could be upregulated by ciglitazone, and ciglitazone-mediated AMPK phosphorylation was essential for stabilizing eNOS expression and activity.

Endothelial nitric oxide synthase phosphorylation promoted nitric oxide synthesis, and nitric oxide-dependent EDR function. We here showed that vaso-relaxation of isolated aortic rings was impaired by ox-LDL stimulation, but effectively restored after ciglitazone pre-treatment. However, AMPK inhibition by compound C further impaired EDR, indicated a critical role of AMPK in regulating ciglitazone-dependent eNOS activity and nitric oxide synthesis. In addition, we also found that ciglitazone induction activated Akt/VEGF pathway and promoted RMVECs-dependent angiogenesis, that might be associated with stabilizing eNOS expression and enhanced eNOS phosphorylation. Previous studies proved the importance of eNOS coupling in AMPK-mediated Akt/VEGF pathway [32,49,50], the mechanism for the role of ciglitazone in AMPK-dependent eNOS coupling and Akt/VEGF signalling would need further investigation.

Ciglitazone, the potent activator of PPARγ, which belonged to a nuclear hormone receptor superfamily, showed the profound effects on endothelium protection against proinflammatory response and oxidative injury through PPARγ-dependent pathway. DCs maturation and infiltration could be suppressed by ciglitazone treatment [13], in which ciglitazone blocked ox-LDL-mediated TLR-4 signalling and TNF-α production *via* PPARγ nuclear translocation [15,16]. Macrophage proliferation and increased cellular cholesteryl esters were also suppressed by ciglitazone, and PPARγ dependent AMPK activation was critically involved [27,51]. PPARγ ligands significantly suppressed high glucose-mediated ICAM-1 expression and inflammation *via* inducing CORM-2 in endothelial cells, which highlighted the perspective in anti-atherogenic drug for diabetic patients [34,52]. However, the protective effect of PPARγ ligands was abolished by pre-treating cells with GW9662, the PPARγ antagonist, or transfecting cells with dominant negative AMPK plasmid [34]. In this study, we found that inhibition of PPARγ with siRNA or GW9662, abolished ciglitazone-induced AMPK phosphorylation, and eNOS-dependent nitric oxide production. In addition, the protective role of ciglitazone in preventing RMVECs ageing and apoptosis, and promoting angiogenesis were also suppressed by PPARγ inhibition. These data indicated that PPARγ activation was required for ciglitazone-mediated endothelial cell survival and function, PPARγ ligands might be effective in therapy against vascular ageing and atherogenesis.

Another important finding in our study was that PPARγ-mediated AMPK activation controlled the LOX-1 expression in RMVECs. A growing body of evidence suggested that oxidative stress-induced LOX-1 was highly associated with eNOS expression [53–55]. Inhibition of LOX-1 by statins induced eNOS synthesis in human coronary artery endothelial cells [38]. In contrast, LOX-1 stimulated by high glucose, high salt or ox-LDL led to a rapid dephosphorylation of eNOS and a subsequent decrease in eNOS activity and expression [23,35]. Consistent with these results, we confirmed that LOX-1 could be regulated by PPARγ at the post-transcriptional level, and ciglitazone-mediated AMPK activation was critically involved. Of note, both AMPK and PPARγ could be regulated by adiponectin receptor-mediated signalling [56,57], and adiponectin induction decreased TNF-α-induced LOX-1 expression [58], which suggested AMPK-PPARγ signalling feedback might protect endothelial cells against atherosclerosis through adjusting cellular oxidative stress stimulated by ox-LDL/LOX-1 axis.

In conclusion, our present study demonstrated that ciglitazone, as a PPARγ agonist, might protect RMVECs against ox-LDL-induced cell apoptosis and ageing, increase nitric oxide synthesis and promote VEGF-dependent angiogenesis through activating AMPK. AMPK activation suppresses LOX-1 expression and increases Akt phosphorylation, which contributes to survival and preserved function of RMVECs. The clarification of PPARγ/AMPK/eNOS pathway would help to find the therapeutic targets for the treatment of endothelial injury and atherosclerosis.

## Supporting information

**Figure S1** *In vitro* cultured RMVECs were stimulated by ox-LDL (50 μg/ml) for 1 hr with or without ciglitazone (10 μmol/l) pre-treatment.

**Figure S2** *In vitro* cultured RMVECs were stimulated by ox-LDL (50 μg/ml) for 1 hr with or without ciglitazone (10 μmol/l) pre-treatment.

**Figure S3** *In vitro* cultured RMVECs were stimulated by ox-LDL (50 μg/ml) for 1 hr with or without ciglitazone (10 μmol/l) pre-treatment.

**Figure S4** Proteins were collected from *in vitro* cultured RMVECs, and the expression of iNOS and eNOS were detected by Western blotting.

**Figure S5** Proteins were collected from *in vitro* cultured RMVECs pre-treated with or without ciglitazone (10 μmol/l), and the expression of iNOS were detected by Western blotting. Representative gel blots depicted the protein level of iNOS.

**Figure S6** Effect of ciglitazone on endothelium-dependent relaxation (EDR) of isolated aortic rings from 8 weeks old male Wistar rats.

**Figure S7** *In vitro* cultured RMVECs were stimulated by ox-LDL (50 μg/ml) for 1 hr with or without LOX-1 siRNA pre-treatment. The expressions of phosphorylated Akt(Ser^473^) and total Akt were examined by Western blotting.
