# Supplementary figures and images for: A protective role of ciglitazone in ox-LDL-induced rat microvascular endothelial cells via modulating PPARγ-dependent AMPK/eNOS pathway
Source: J Cell Mol Med. 2014 Nov 11;19(1):92–102. doi: 10.1111/jcmm.12463 (PMC4288353; doi:10.1111/jcmm.12463)

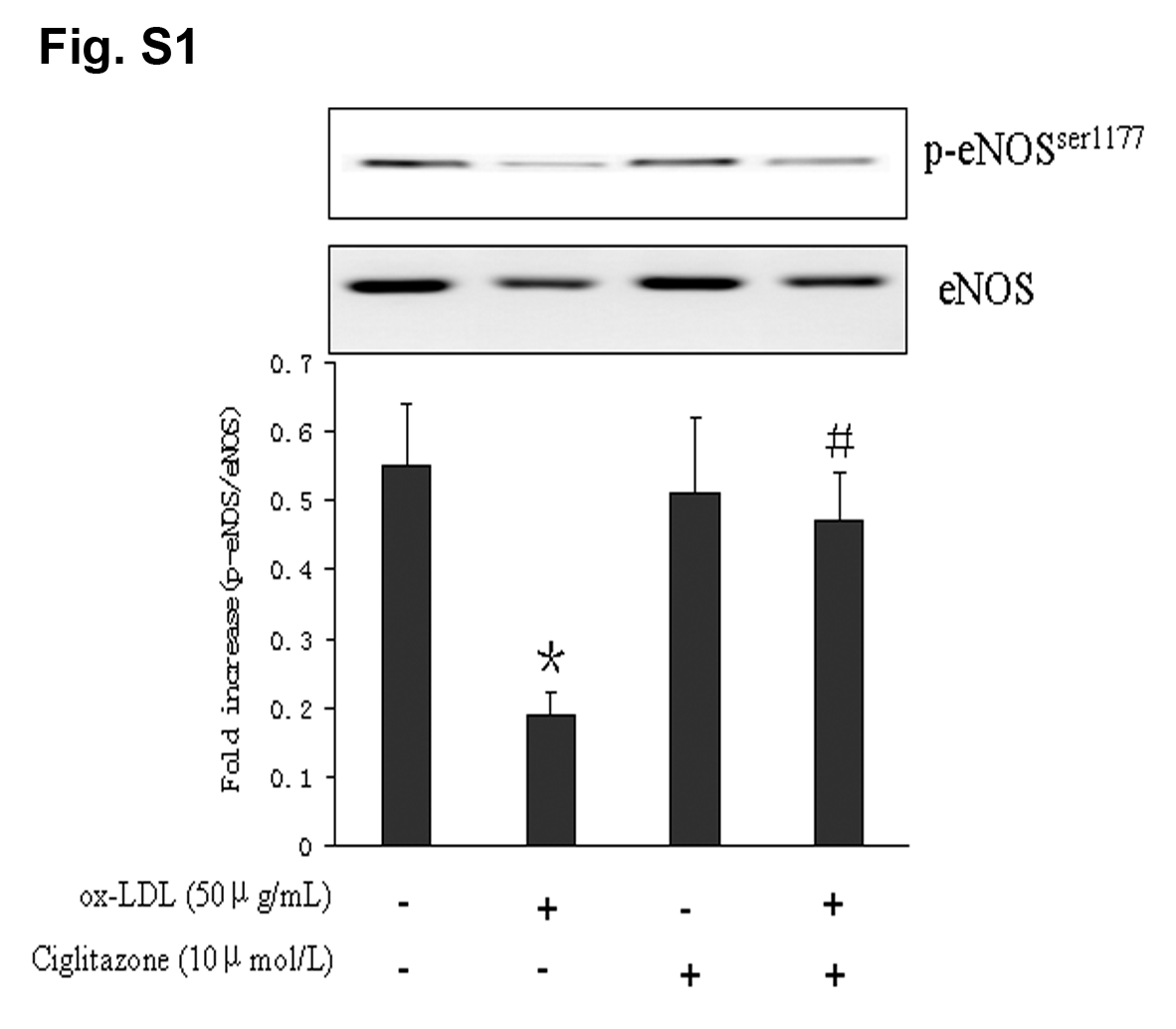

Supplement: Supplementary file 1 — Figure S1 In vitro cultured RMVECs were stimulated by ox-LDL (50 μg/ml) for 1 hr with or without ciglitazone (10 μmol/l) pre-treatment. [file jcmm0019-0092-sd1.tif]

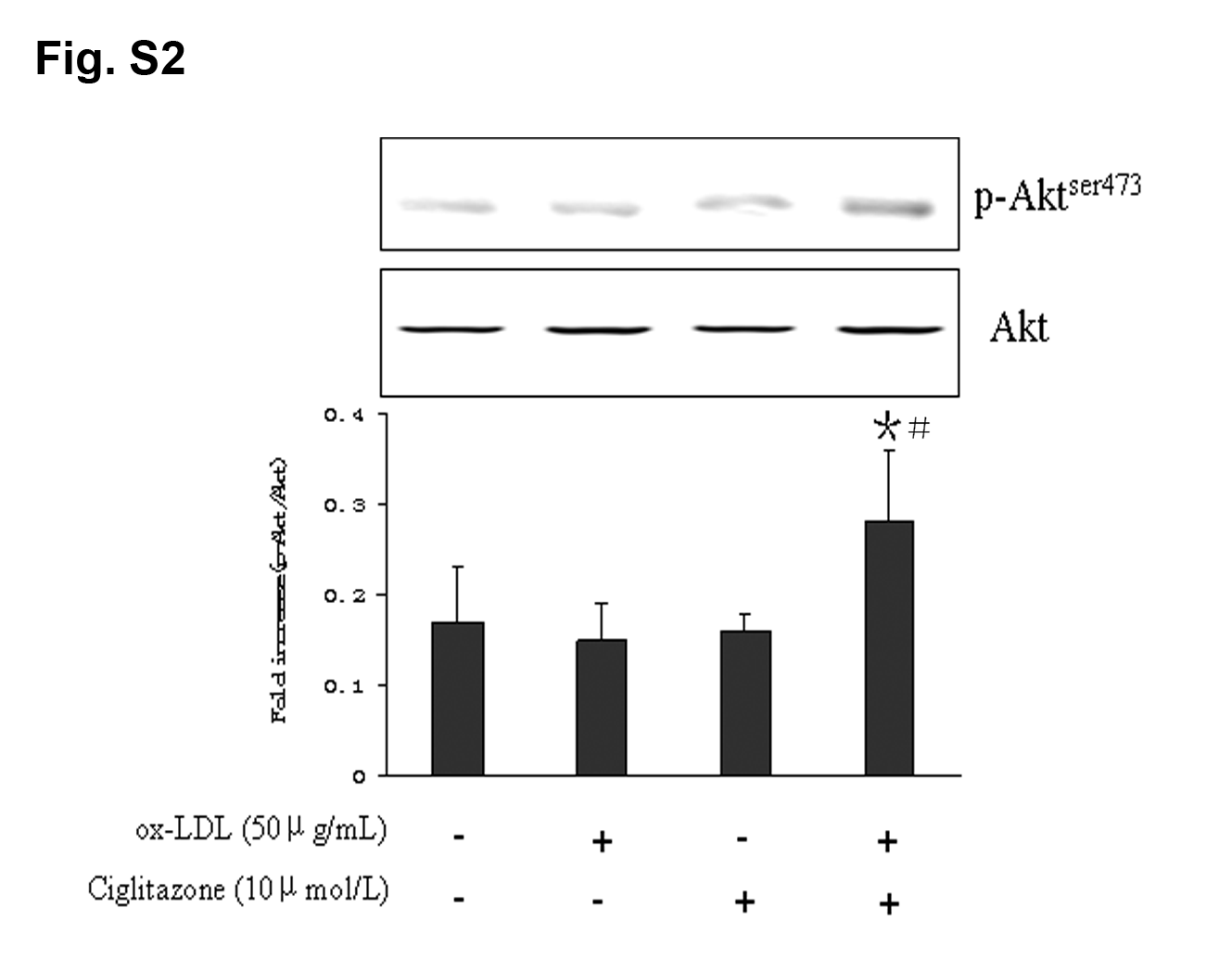

Supplement: Supplementary file 2 — Figure S2 In vitro cultured RMVECs were stimulated by ox-LDL (50 μg/ml) for 1 hr with or without ciglitazone (10 μmol/l) pre-treatment. [file jcmm0019-0092-sd2.tif]

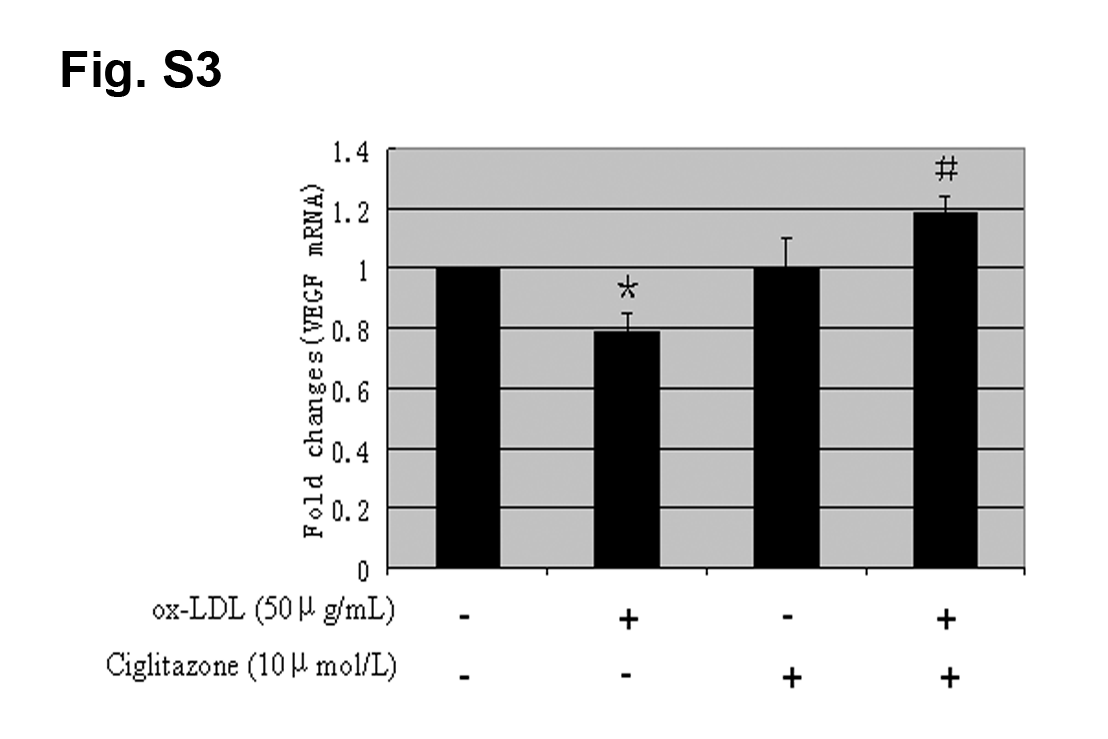

Supplement: Supplementary file 3 — Figure S3 In vitro cultured RMVECs were stimulated by ox-LDL (50 μg/ml) for 1 hr with or without ciglitazone (10 μmol/l) pre-treatment. [file jcmm0019-0092-sd3.tif]

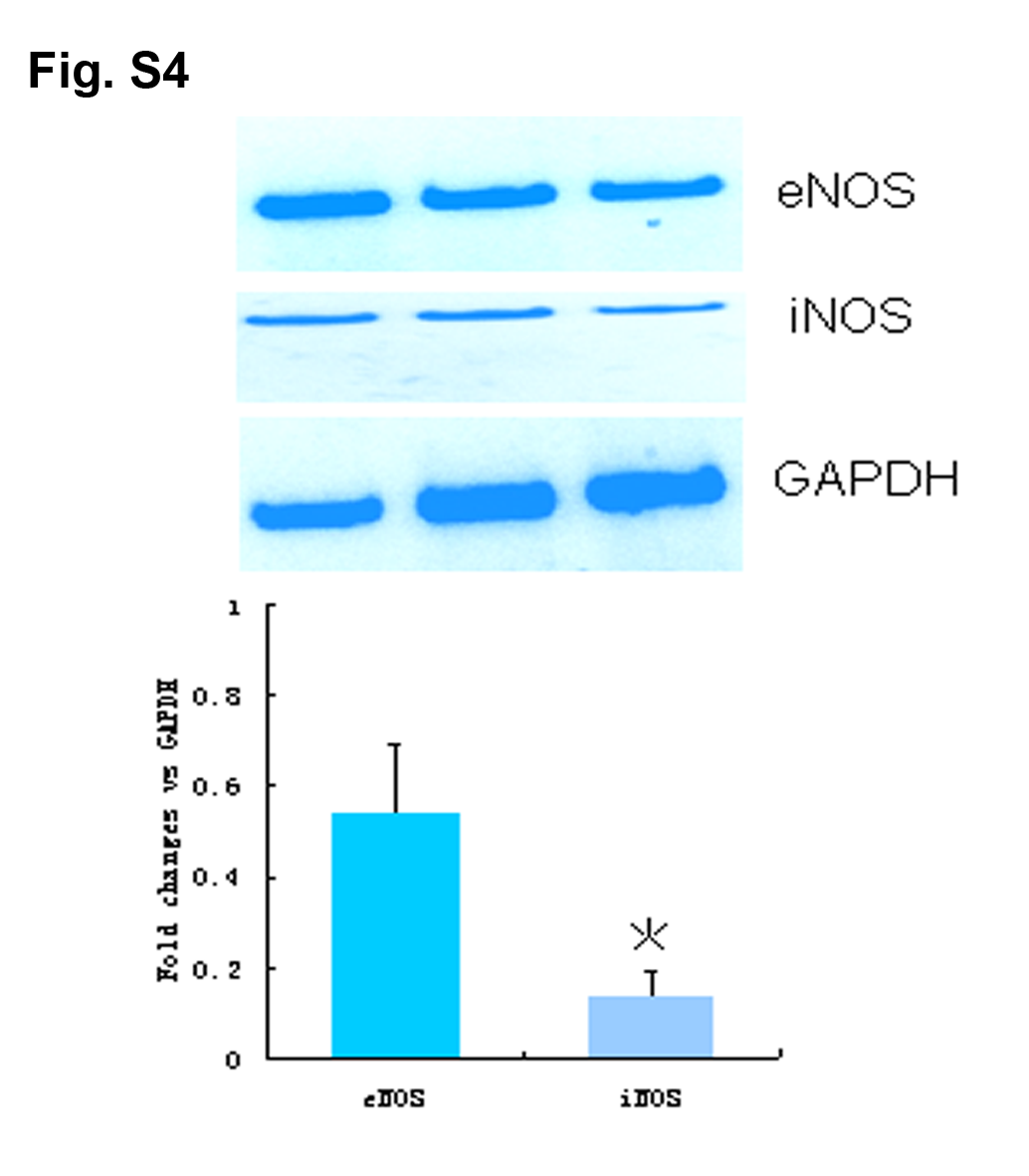

Supplement: Supplementary file 4 — Figure S4 Proteins were collected from in vitro cultured RMVECs, and the expression of iNOS and eNOS were detected by Western blotting. [file jcmm0019-0092-sd4.tif]

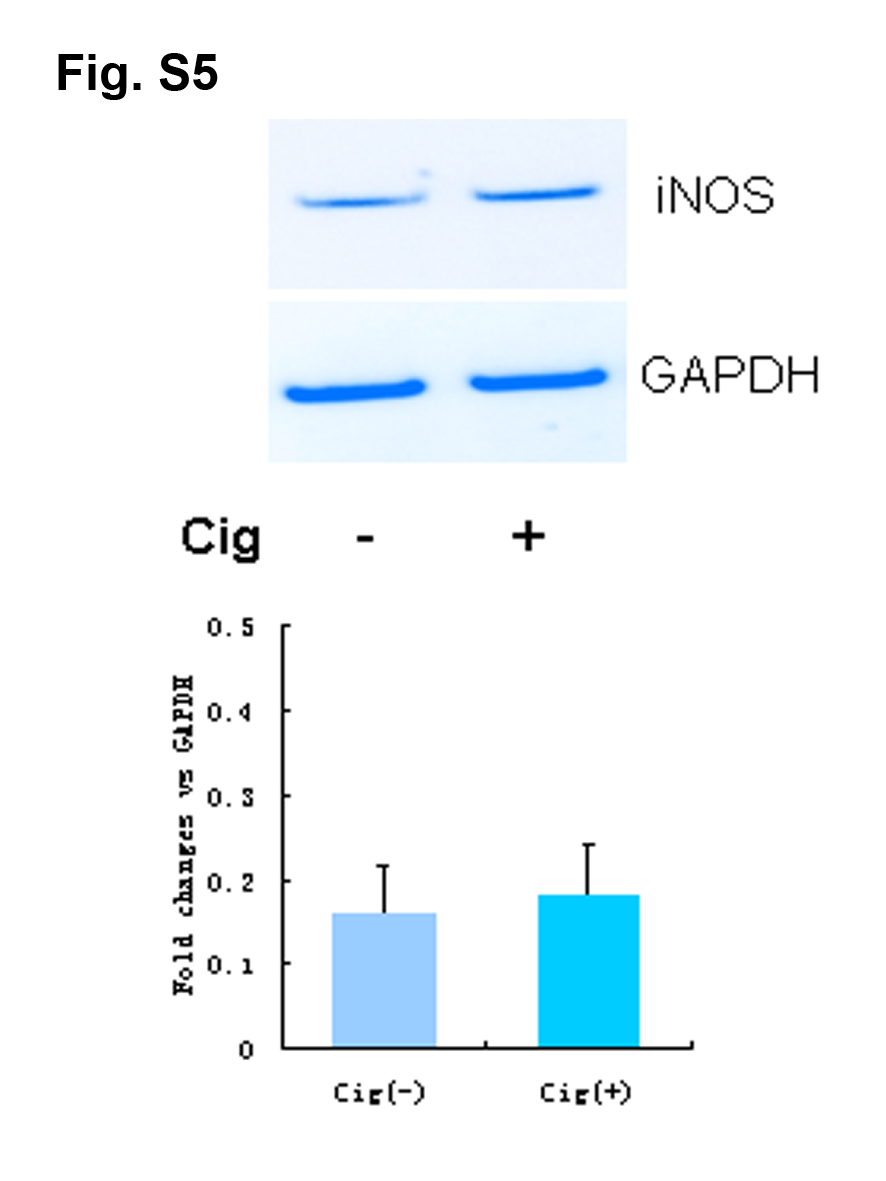

Supplement: Supplementary file 5 — Figure S5 Proteins were collected from in vitro cultured RMVECs pre-treated with or without ciglitazone (10 μmol/l), and the expression of iNOS were detected by Western blotting. Representative gel blots depicted the protein level of iNOS. [file jcmm0019-0092-sd5.tif]

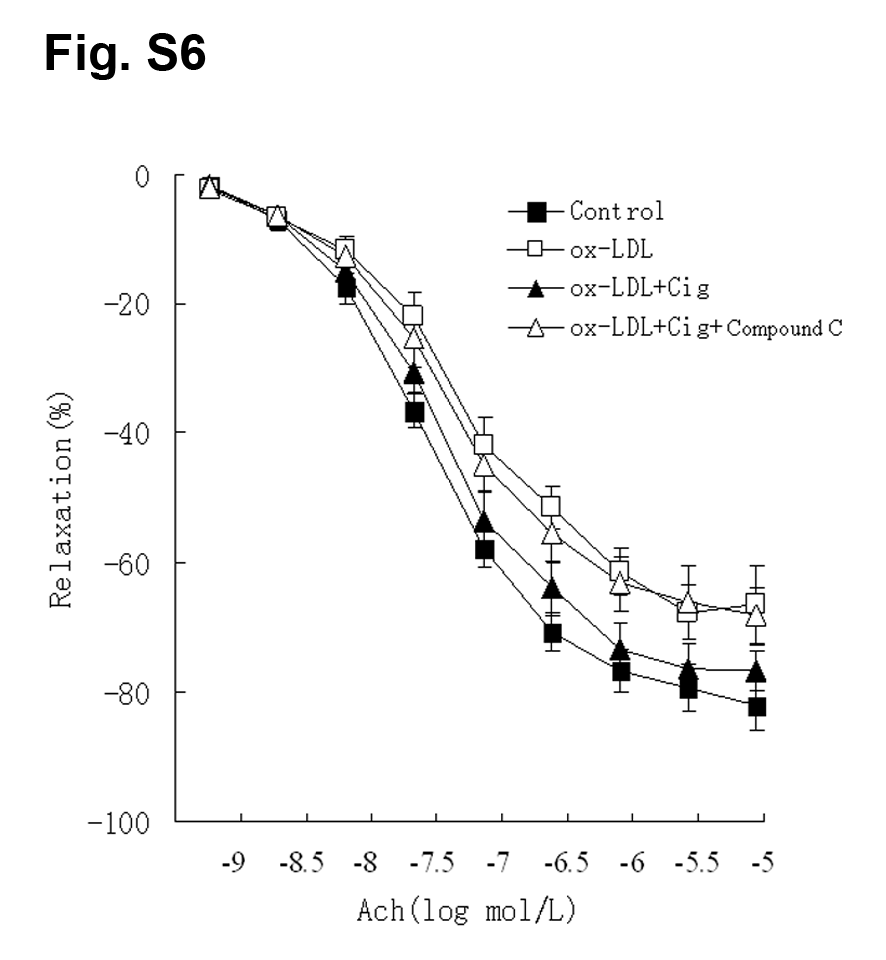

Supplement: Supplementary file 6 — Figure S6 Effect of ciglitazone on endothelium-dependent relaxation (EDR) of isolated aortic rings from 8 weeks old male Wistar rats. [file jcmm0019-0092-sd6.tif]

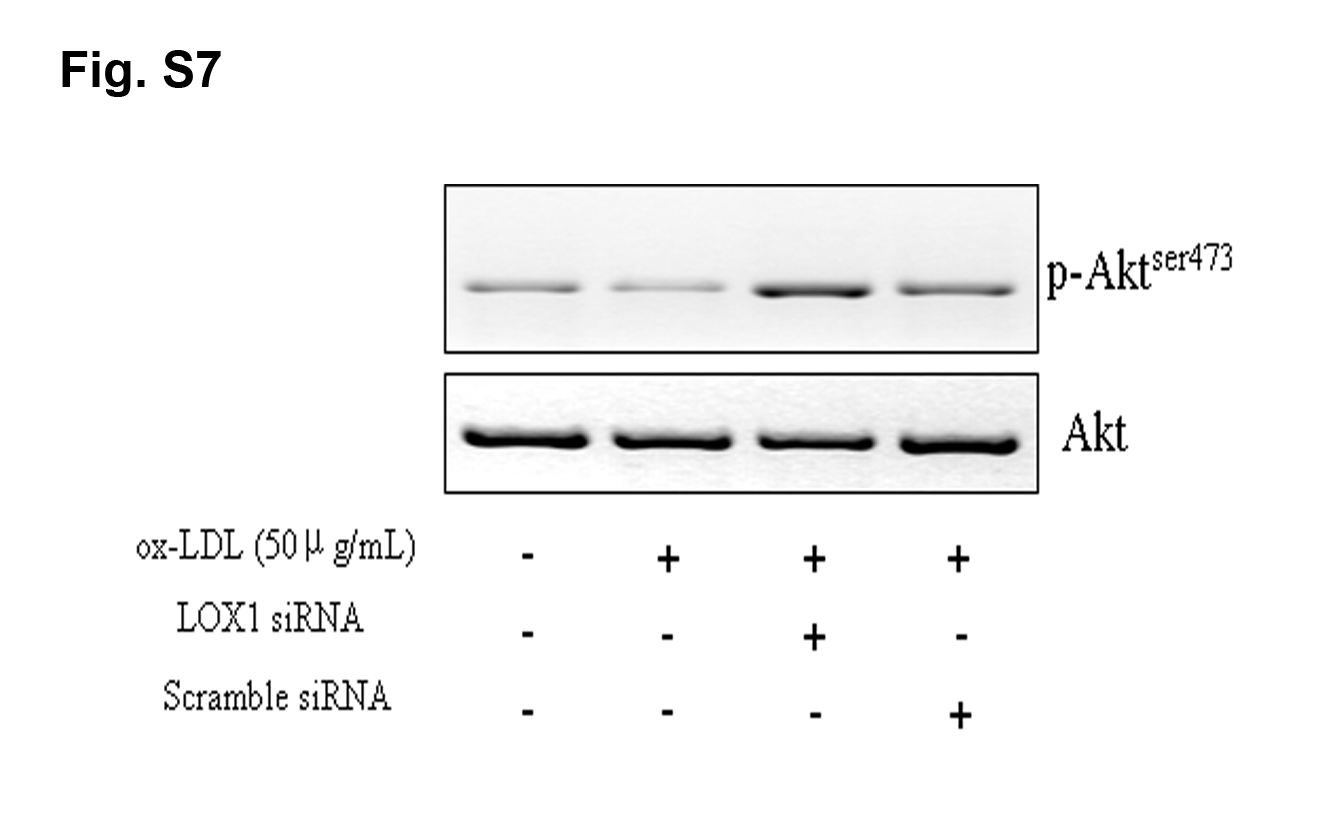

Supplement: Supplementary file 7 — Figure S7 In vitro cultured RMVECs were stimulated by ox-LDL (50 μg/ml) for 1 hr with or without LOX-1 siRNA pre-treatment. The expressions of phosphorylated Akt(Ser473) and total Akt were examined by Western blotting. [file jcmm0019-0092-sd7.tif]
